# Adaptive Deformation Control for Elastic Linear Objects

**DOI:** 10.3389/frobt.2022.868459

**Published:** 2022-04-28

**Authors:** Omid Aghajanzadeh, Miguel Aranda, Juan Antonio Corrales Ramon, Christophe Cariou, Roland Lenain, Youcef Mezouar

**Affiliations:** ^1^ CNRS, Clermont Auvergne INP, Institut Pascal, Université Clermont Auvergne, Clermont-Ferrand, France; ^2^ Université Clermont Auvergne, INRAE, URTSCF, Clermont-Ferrand, France; ^3^ Instituto de Investigación en Ingeniería de Aragón, Universidad de Zaragoza, Zaragoza, Spain; ^4^ Centro Singular de Investigación en Tecnoloxías Intelixentes (CiTIUS), Universidade de Santiago de Compostela, Santiago de Compostela, Spain

**Keywords:** deformable linear object, robot manipulation, adaptive control, tracking of deformation, parameters estimation, Lyapunov stability

## Abstract

This paper addresses the general problem of deformable linear object manipulation. The main application we consider is in the field of agriculture, for plant grasping, but may have interests in other tasks such as human daily activities and industrial production. We specifically consider an elastic linear object where one of its endpoints is fixed, and another point can be grasped by a robotic arm. To deal with the mentioned problem, we propose a model-free method to control the state of an arbitrary point that can be at any place along the object’s length. Our approach allows the robot to manipulate the object without knowing any model parameters or offline information of the object’s deformation. An adaptive control strategy is proposed for regulating the state of any point automatically deforming the object into the desired location. A control law is developed to regulate the object’s shape thanks to the adaptive estimation of the system parameters and its states. This method can track a desired manipulation trajectory to reach the target point, which leads to a smooth deformation without drastic changes. A Lyapunov-based argument is presented for the asymptotic convergence of the system that shows the process’s stability and convergence to desired state values. To validate the controller, numerical simulations involving two different deformation models are conducted, and performances of the proposed algorithm are investigated through full-scale experiments.

## 1 Introduction

Deformable object manipulation has many applications in human life, including domestic chores, industry, medical tasks, food handling, and agriculture. However, it is very hard to manipulate these objects effectively with robots. It is indeed difficult to model them accurately and precisely predict their deformation (Sanchez et al., 2018). In addition, one of the biggest challenges in controlling the shape of deformable objects is their infinite degrees of freedom.

Some works related to manipulating deformable objects have been done by considering an accurate object model. Mass-spring models (Tokumoto et al., 1999), finite element methods (Koch et al., 1996; Keeve et al., 1998), and boundary element methods (James and Pai, 2003) are found repeatedly in the literature. On the contrary, several recent works do not need an exact deformable object model by using some approximations of the deformation behavior (Navarro-Alarcon and Liu, 2017; Hu et al., 2018; Zhu et al., 2018; Cherubini et al., 2020; Zhu et al., 2021b). These models are linear and can be computed in real-time with a small number of data (Zhu et al., 2022). They need to be continuously updated during the object deformation since they are local.

The controlled manipulation of deformable objects is a challenging problem that requires sophisticated algorithms. Jeon and Choi (2007) reported a locally optimal method to control the behavior of physically-based simulation of deformable objects. Aranda et al. (2019) is focused on the coordination of multiple robots while manipulating an object, minimizing a measure of deformation. Ding et al. (2018) proposed a dynamic surface control strategy to control a deformable linear object by trying to eliminate the vibration. These methods cannot control the trajectory of the manipulation of the deformable objects since they were designed to reach fixed set-points. Navarro-Alarcon and Liu (2017) used an adaptive estimation of a deformation Jacobian to design a robotic shape controller of a deformable object. Navarro-Alarcon and Liu (2014) used an online estimation method to control the shape of the objects. However, the performance of the method presented by Navarro-Alarcon and Liu (2014) necessitates a prior-known structure and depends on the choice of regression matrix. A vision-based method that computes parameterized regression features to reshape an elastic rod is presented in Qi et al. (2020). Similarly, Lagneau et al. (2020) proposed a model-free visual servoing approach for deforming a wire into a desired shape. These algorithms are used for low-speed movements and they need prior knowledge about the deformation of the object or to perceive the entire shape of the object during the manipulation to calculate the used deformation Jacobian.

This paper focuses on an elastic linear rod manipulation with one of its ends fixed, with agriculture as the main targeted field of application. Many agriculture tasks need to reshape objects (Davenport, 2006; He and Schupp, 2018; Zhang et al., 2018). In fact, most agriculture elements (such as branches) can be assumed as deformable linear objects. In this field, skilled labor is scarce, and labor costs are increasing significantly. Meanwhile, worker safety is another issue in traditional farming. Therefore mechanical or robotic solutions for reducing the amount of hand labor seem necessary (He and Schupp, 2018). The problem is that there are few works about the manipulation of deformable objects in this domain, and the existing deformable object manipulation approaches cannot be applied directly in this field. Accordingly, this paper aims to develop a method to be used in the agricultural field for some tasks such as pruning the trees. Pruning is the most common tree maintenance procedure. Pruning must be done correctly because improper pruning can create lasting damage or shorten the tree’s life. In this task, the main point is to deform the branches so that the cutting point moves to where the branch can be cut appropriately. The proposed approach can also be helpful to manipulate the stem or branches of a vegetable plant for plant inspection and fruit harvesting tasks.

As we mentioned, most of the proposed methods in the literature need some information about the object (an exact model or an estimation of the deformation). They mostly need to perceive the total shape of the object during the deformation process. Therefore, applying these methods with these limitations to the objects that exist in the agriculture field is not easy. This inspired us to develop a method to solve these issues and deform the object while moving a point on the object to a specific target without causing any breakage. Our method does not need to perceive the entire shape of the object and does not attach to any particular model or object. Therefore, in this study, we propose an adaptive control strategy, using a gripper grasping the object at a given point to regulate the state (position and orientation) of an arbitrary point on the body of the object. The proposed method does not need prior knowledge of any model parameters of the object and works in real-time. There are several contributions in our work:

• We take as starting point an adaptive control scheme proposed for biomedical applications (Aghajanzadeh et al., 2017, 2018) and we extend it to a novel problem which is deformable object manipulation.
• To the best of our knowledge, the existing adaptive approaches (Navarro-Alarcon et al., 2014; Navarro-Alarcon and Liu, 2017; Zhu et al., 2018; Lagneau et al., 2020) simply reach a fixed set-point without considering any deformation trajectory while our controller can be used to track a desired dynamic evolution of the state. This helps to have a closer control on the manipulation of the object and time of the task’s completion.
• Compared with (Navarro-Alarcon et al., 2014), one does not need to calculate the Jacobian to find the relationship between the deformation of the object and motion of the gripper using the current method. The presented controller also does not need offline information of the object’s deformation.
• Compared with (Navarro-Alarcon et al., 2014; Li et al., 2019), the current method can control the full states of the controlled point, including the angle.
• Compared with (Navarro-Alarcon and Liu, 2017; Zhu et al., 2018), our work contains a formal analysis of the system dynamics under the controller.

Even though our method does not control the full shape of the object, controlling as we do the position and orientation at one point is sufficient for some tasks, requires to perceive online only a single point (not the full shape of the object), and implies the system is fully actuated, which can reduce the impact of local minima. One of the most interesting properties of our method is that it can be successfully applied on objects with fairly diverse characteristics. To demonstrate this, we provide an extensive validation including simulation tests with different deformation models (Kirchhoff rod, As-Rigid-As-Possible), and real robotic experiments with varied objects: a sponge rod, a plexiglass rod, and real vegetable plants^1^.

### 1.1 Notation

Vectors and matrices are presented by capital letters, e.g., *M* ∈ *R* ^*g*×*h*^. Parameters and variables are denoted by lower-case letters, e.g., *p*_1_.

## 2 Problem Definition

**FIGURE 1:**
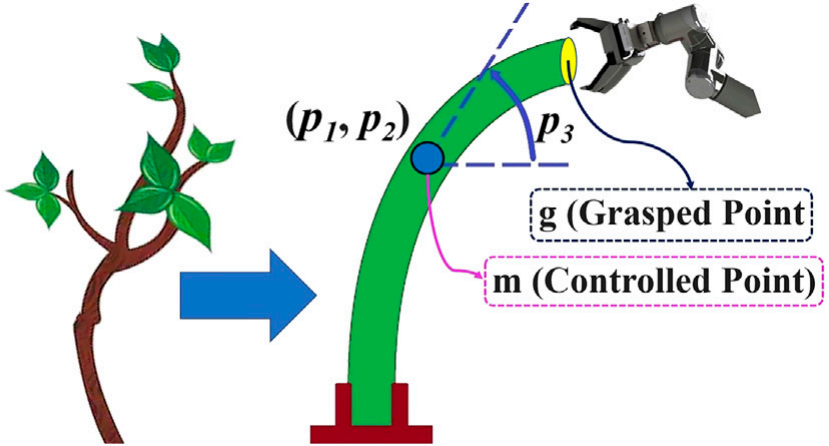
Considered object in the current study.

This section defines the challenge we deal with in this study. We consider elastic linear objects, illustrated schematically in Figure 1. The assumptions of our work are as follows. We assume that the linear object lies in a 2-D workskpace, which is still a complex and interesting challenge (Koessler et al., 2021; Zhu et al., 2018). The object is represented as a continuous curve and its state by the position and orientation (tangent angle of the curve) at each point along its length. One of the ends is fixed, and the rest of the object is free to move. The length of the rod is denoted as *L*. We assume that the object deforms elastically and its size fits the workspace of the robotic arm. A robotic gripper is used to grasp rigidly the other end (which we call the grasped point *g*) on the object, and it can set the object’s state at that point. Moreover, we assume that the shape of the object always stays in quasi-static stable equilibrium, and as shown in Figure 1, the whole shape of the object can be manipulated by controlling the motion of *g*. It should also be noted that the thickness range of the object is dependent on the opening-closing range of the gripper. A camera is used to track the pose of the controlled point (*m*), and it is assumed that the position and orientation of *m* can be known during the deformation process. The exact position and orientation of the target point are known before the start of the procedure, and they are defined in the reachable range of the robot workspace where the object can be reach (i.e., the manipulation task is feasible).

The problem we address is the control of the state of an arbitrarily chosen point (*m*) on the body of the object using *g*, and the manipulation task is to move *m* to the target position and orientation *M*_*f*_. *M* denotes the state of the controlled point *m*, and *G* denotes the state of the grasped point *g*, which are defined as:

$$M={\left[p_{1m}\;p_{2m}\;p_{3m}\right]}^{T},\;G={\left[p_{1g}\;p_{2g}\;p_{3g}\right]}^{T}$$ (1)

The main difficulty is that solving this indirect control problem requires taking into account the deformation behavior of the object appropriately. The controller is indirect since we control a point on the body of the object using another point (which is the grasped point). To solve the problem, we will design an indirect adaptive controller, to be discussed later in the paper.

## 3 Controller Design

In this section, an adaptive control method to manipulate a point along the object’s length by moving the gripper is described. Our objective is to develop a controller to reshape the object so that *m* reaches its target configuration without the need to know any model parameters. The final state of the target point is denoted by $M_{f}={\left[p_{1mf},p_{2mf},p_{3mf}\right]}^{T}$ . Like (Navarro-Alarcon and Liu, 2017; Zhu et al., 2018; Hu et al., 2019; Lagneau et al., 2020; Yu et al., 2021), the control input is specified as:

$$U=\dot{G}$$ (2)

To control the linear deformable objects properly, we try to set an appropriate relationship between the motion of the gripper and the controlled point. In this context, we were inspired by (Ogden, 1997; Navarro-Alarcon et al., 2014) which explained that a relationship exists between *G* and *M*:

$$M=f_{1}\left(\dot{G}\right)\;\;\;\;or\;\;\;\dot{M}=f_{2}\left(\dot{G}\right)$$ (3)

Accordingly, for the motion of *g*, one can write:

$$\dot{G}=F\left(M,\dot{M}\right)$$ (4)

In the current study, the objective is to design a control law for *U* $\left({\left[u_{1},\;u_{2},\;u_{3}\right]}^{T}\right)$ . However, we emphasize that the mentioned existing works can only control the instantaneous variation of the state of the controlled variables. By doing so, they can make the state reach a fixed set-point. In this work, we introduce additional terms in the structure of the controller. These terms allow us to track a desired trajectory of the state, instead of just reaching a fixed set-point. The suitability of the controller formulation that we propose is supported by the fact that it follows well-known adaptive control techniques (Slotine and Li, 1991) and previous works in other application contexts (Aghajanzadeh et al., 2017, 2018). Hence, in this study, the relationship between the grasped point and the controlled point is assumed as follows:

$$\dot{G}=\lambda \dot{M}+\Omega \tilde{M}$$ (5)

where Ω is a 3 × 3 matrix that consists of some unknown parameters we named *a*_*ij*_ (*i* & *j* = 1, 2, 3) and $\tilde{M}$ is *M* − *M*_*des*_. *M*_*des*_(*t*) is the desired trajectory of *M*. This trajectory is defined as bounded and differentiable. Our goal is to design an adaptive indirect controller to track this trajectory. Note that we use the desired state variable *M*_*des*_ in the dynamic Eq. 5 to reach the target point in a limited time based on a desired deformation trajectory. *λ* is then defined as a vector with positive components [*a*_10_, *a*_20_, *a*_30_], which permits to account for the speed of the controlled point *m* during the process. Therefore, according to Eq. 5, one can write the following relationship between the motion of *m* and *g*:

$${\dot{p}}_{ig}=a_{i0}{\dot{p}}_{im}+\overset{3}{\underset{j=1}{\sum }}a_{ij}{\tilde{p}}_{jm}$$ (6)

where the tracking errors ${\tilde{p}}_{im}$ are *p*_*im*_ − *p*_*imdes*_. We introduce *p*_*imdes*_ as the desired state values of the controlled point *m*, which are time-varying to track a desired trajectory of the state by the adaptive controller. They will be defined later. *a*_*ik*_ (*k* = 0, *j*) are dependent on the current shape of the object. It is reasonable to assume these parameters are approximately constant in a local neighborhood of the current operating point (i.e., current shape) since they change smoothly. As a result, to build a control based on this model, these parameters have to be estimated and online updated. An adaptive estimation algorithm is here introduced to estimate these parameters.

### 3.1 Adaptive Estimation Algorithm

**FIGURE 2:**
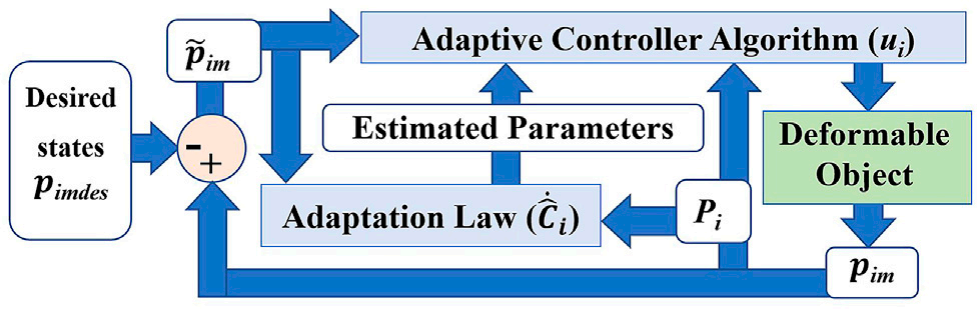
Schematic diagram of the proposed adaptive control strategy.

In this section, the adaptive control strategy is developed for Eq. 6. Figure 2 shows the block diagram of the proposed controller.

First, we rearrange the right-hand side of Eq. 6.

$$a_{i0}{\dot{p}}_{im}+\overset{3}{\underset{j=1}{\sum }}a_{ij}{\tilde{p}}_{jm}=R_{i}C_{i}^{T}$$ (7)

where:

$$R_{i}=\left[{\dot{p}}_{im}\;{\tilde{p}}_{1m}\;{\tilde{p}}_{2m}\;{\tilde{p}}_{3m}\right]$$ (8)

$$C_{i}=\left[a_{i0}\;a_{i1}\;a_{i2}\;a_{i3}\right]$$ (9)

With the purpose of finding the estimation of *a*_*ik*_, the adaptive control law $\left({\left[u_{1},\;u_{2},\;u_{3}\right]}^{T}\right)$ is defined as:

$$u_{i}={\hat{a}}_{i0}\left({\dot{p}}_{imdes}-\alpha_{i}{\tilde{p}}_{im}\right)+\overset{3}{\underset{j=1}{\sum }}{\hat{a}}_{ij}{\tilde{p}}_{jm}$$ (10)

The goal of this controller is to set the speed of *m* with the proposed forms $\left({\dot{p}}_{imdes}-\alpha_{i}{\tilde{p}}_{im}\right)$ , which will help us to generate desired trajectories for the velocities to be used in the controller design. *α*_*i*_ are some positive constants. The accent *ˆ* is used for the estimated parameters of the system. In other words, ${\hat{a}}_{ik}$ are some estimates of the time-varying parameters *a*_*ik*_ that will be updated using an adaptation law. They are the estimations of the true parameters *a*_*ik*_.

By rearranging Eq. 10, the control law can be expressed as:

$$u_{i}=P_{i}{\hat{C}}_{i}^{T}$$ (11)

where:

$$P_{i}=\left[\left({\dot{p}}_{imdes}-\alpha_{i}{\tilde{p}}_{im}\right)\;{\tilde{p}}_{1m}\;{\tilde{p}}_{2m}\;{\tilde{p}}_{3m}\right]$$ (12)

The vectors of system parameters’ estimations are:

$${\hat{C}}_{i}=\left[{\hat{a}}_{i0}\;{\hat{a}}_{i1}\;{\hat{a}}_{i2}\;{\hat{a}}_{i3}\right]$$ (13)

The adaptation law for updating the estimated parameters is defined as:

$${\dot{\hat{C}}}_{i}=-P_{i}T_{i}{\tilde{p}}_{im}$$ (14)

The adaptation gains *T*_*i*_ are constant non-singular positive definite matrices of size 4 × 4. Note that, interestingly, the information that needs to be measured to implement the proposed adaptive feedback controller is just the value of *M* at each instant of time. The controller will be analyzed in the next section using the Lyapunov formalism.

**Proposition**: Under the action of the proposed control law *u*_*i*_ Eq. 10, the control system tracks the desired trajectory, i.e., ${\tilde{p}}_{im}\to 0$ as *t* → *∞* for *i* = 1, 2, 3.

**Proof**: The closed-loop dynamics of the system using the proposed controller are obtained in this part. For this purpose, the control law Eq. 10 is substituted in Eq. 6, and by adding and subtracting a term, we have:

$$a_{i0}{\dot{p}}_{im}+\overset{3}{\underset{j=1}{\sum }}a_{ij}{\tilde{p}}_{jm}={\hat{a}}_{i0}\left({\dot{p}}_{imdes}-\alpha_{i}{\tilde{p}}_{im}\right)+\overset{3}{\underset{j=1}{\sum }}{\hat{a}}_{ij}{\tilde{p}}_{jm}+a_{i0}\left({\dot{p}}_{imdes}-\alpha_{i}{\tilde{p}}_{im}\right)-a_{i0}\left({\dot{p}}_{imdes}-\alpha_{i}{\tilde{p}}_{im}\right)$$ (15)

By rearranging Eq. 15, we have:

$$a_{i0}\left({\dot{p}}_{im}-\left({\dot{p}}_{imdes}-\alpha_{i}{\tilde{p}}_{im}\right)\right)=\left({\dot{p}}_{imdes}-\alpha_{i}{\tilde{p}}_{im}\right)\left({\hat{a}}_{i0}-a_{i0}\right)+\overset{3}{\underset{j=1}{\sum }}{\tilde{p}}_{jm}\left({\hat{a}}_{ij}-a_{ij}\right)$$ (16)

According to Eqs. 12, 13, 16, the system’s closed-loop dynamics for every *i* (*i* = 1, 2, 3) is found as:

$$a_{i0}\left({\dot{\tilde{p}}}_{im}+\alpha_{i}{\tilde{p}}_{im}\right)=P_{i}{\tilde{C}}_{i}^{T}$$ (17)

note that ${\tilde{C}}_{i}={\hat{C}}_{i}-C_{i}$ . By rearranging Eq. 17, the dynamics are reformulated as:

$${\dot{\tilde{p}}}_{im}=-\alpha_{i}{\tilde{p}}_{im}+\frac{1}{a_{i0}}P_{i}{\tilde{C}}_{i}^{T}$$ (18)

A positive definite Lyapunov function candidate is used next to analyze the system stability and the tracking convergence using the proposed controller.

$$V=\frac{1}{2}\left(\overset{3}{\underset{i=1}{\sum }}{\tilde{p}}_{im}^{2}+\frac{1}{a_{i0}}{\tilde{C}}_{i}T_{i}^{-1}{\tilde{C}}_{i}^{T}\right)\geq 0$$ (19)

The time derivative of *V* is obtained as:

$$\dot{V}=\overset{3}{\underset{i=1}{\sum }}\left({\tilde{p}}_{im}{\dot{\tilde{p}}}_{im}+\frac{1}{a_{i0}}{\dot{\tilde{C}}}_{i}T_{i}^{-1}{\tilde{C}}_{i}^{T}\right)$$ (20)

We can write ${\dot{\tilde{C}}}_{i}={\dot{\hat{C}}}_{i}$ since ${\dot{C}}_{i}$ is negligible compared to ${\dot{\hat{C}}}_{i}$ . Consequently, via the closed-loop dynamics Eqs. 18, 20 can be expressed as:

$$\dot{V}=\overset{3}{\underset{i=1}{\sum }}\left(-\alpha_{i}{\tilde{p}}_{im}^{2}+\frac{1}{a_{i0}}P_{i}{\tilde{C}}_{i}^{T}{\tilde{p}}_{im}+\frac{1}{a_{i0}}{\dot{\tilde{C}}}_{i}T_{i}^{-1}{\tilde{C}}_{i}^{T}\right)$$ (21)

Using the parameters’ adaptation law Eq. 14, the time derivative of Lyapunov function Eq. 21 is simplified to:

$$\dot{V}=\overset{3}{\underset{i=1}{\sum }}-\alpha_{i}{\tilde{p}}_{im}^{2}$$ (22)

As one can see, the time derivative of the Lyapunov function is negative semi-definite.

The Lyapunov function proposed in Eq. 19 is positive definite (*V*(*t*) > 0) in terms of ${\tilde{p}}_{im}$ and ${\tilde{C}}_{i}$ . Its time derivative is negative semi-definite $\left(\dot{V}\leq 0\right)$ ; therefore, *V*(*t*) is bounded. Accordingly, one can conclude that ${\tilde{p}}_{im}$ and ${\tilde{C}}_{i}$ remain bounded. In addition, the desired trajectories of the states $\left({\tilde{p}}_{imdes}\right)$ are defined bounded; thus, the boundedness of $p_{im}=\left(p_{imdes}+{\tilde{p}}_{im}\right)$ is also concluded. Now, the function *b*(*t*) is defined:

$$b\left(t\right)=\overset{3}{\underset{i=1}{\sum }}\alpha_{i}{\tilde{p}}_{im}^{2}\geq 0$$ (23)

such that $b\left(t\right)=-\dot{V}\left(t\right)\geq 0$ , whose integration gives:

$$V\left(0\right)-V\left(\infty \right)=\underset{t\to \infty }{\lim}\int_{0}^{t}b\left(\eta \right)d\eta$$ (24)

Considering that $\dot{V}=dV/dt$ is negative semi-definite, (*V* (0) − *V* (*∞*)) is positive and finite. Therefore, $\lim_{t\to \infty }\int_{0}^{t}b\left(\eta \right)d\eta$ has a finite positive value. Moreover, according to the Barbalat’s lemma (Slotine and Li, 1991), if *b*(*t*) is uniformly continuous and the limit of integral $\lim_{t\to \infty }\int_{0}^{t}b\left(\eta \right)d\eta$ exists, then:

$$\underset{t\to \infty }{\lim b\left(t\right)}=0$$ (25)

Based on Eq. 23 and considering Eq. 25, it is concluded that:

$$\underset{t\to \infty }{\lim}\left(\overset{3}{\underset{i=1}{\sum }}\alpha_{i}{\tilde{p}}_{im}^{2}\right)=0$$ (26)

It is known that *α*_*i*_ > 0 are non-zero positive constants and ${\tilde{p}}_{im}^{2}\geq 0$ are positive. As a result, Eq. 26 implies that ${\tilde{p}}_{im}\to 0$ as *t* → *∞*. Consequently, the states of the controlled points converge to their corresponding desired values (*p*_*im*_ → *p*_*imdes*_).

## 4 Simulation Results

To show that our proposed control approach is generic, we have chosen two different simulated deformation models in our tests. To evaluate the proposed adaptive control strategy, the controller Eq. 11 and the adaption law for updating the estimated parameters Eq. 14 are simulated. At each time instant (*t*), using Eq. 11 the position and orientation of the grasped point are updated, and the new shape of the object is obtained using the simulated deformation models. It is assumed that the rod’s shape remains stable during the entire control process. Intending to obtain a smooth and uniform dynamic evolution of the object’s shape, we define exponential desired evolutions of the state. These allow reaching the desired final state of the controlled point in a limited time. Specifically, the following desired states are intended to be tracked using the proposed control strategy:

$$p_{ides}=\left(p_{im0}-p_{imf}\right)\;exp\left(-g_{i}t\right)+p_{imf}$$ (27)

*p*_*im0*_ are the initial values of *M*. The parameters *g*_*i*_ (the exponential coefficient) are positive constants.

### 4.1 Simulations Using Kirchhoff Elastic Rod Model

**FIGURE 3:**
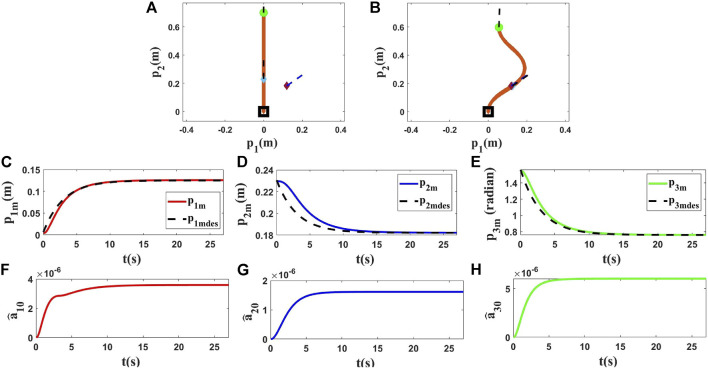
Simulation 1. First row: **(A)** and **(B)** are the initial and final shape of the object, respectively. The desired state of the point *m* is shown as a purple diamond (position) and a dashed line segment (orientation). *L* is 0.70(*m*), *M* at time zero is *M*_0_ = [0.0000, 0.2310, 1.5708]^*T*^, and *M*_*fdes*_ = [0.1200, 0.1820, 0.7600]^*T*^. After applying the proposed controller, the final state of *M* is *M*_*f*_ = [0.1206, 0.1820, 0.7610]^*T*^. Second row: **(C)**, **(D)** and **(E)** show the states of the controlled point regarding their desired trajectories. Third row: **(F)**, **(G)** and **(H)** show ${\hat{a}}_{10}$ , ${\hat{a}}_{20}$ and ${\hat{a}}_{30}$ in the first simulation using the Kirchhoff model.

**FIGURE 4:**
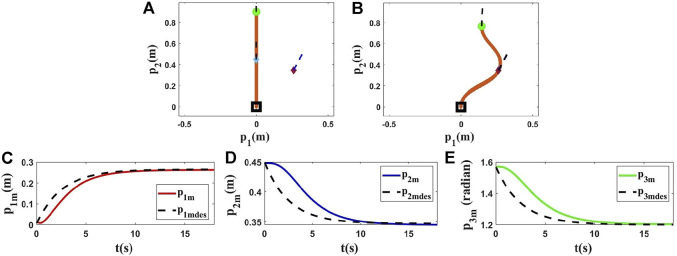
Simulation 2. First row: **(A)** and **(B)** are the initial and final shape of the object, respectively. The desired state of the point *m* is shown as a purple diamond (position) and a dashed line segment (orientation). *L* is 0.90(*m*), *M*_0_ = [0.0000, 0.4500, 1.5708]^*T*^, and *M*_*fdes*_ = [0.2600, 0.3470, 1.2000]^*T*^. After applying the proposed controller, the final state of *M* is *M*_*f*_ = [0.2600, 0.3467, 1.2000]^*T*^. Second row: **(C)**, **(D)** and **(E)** are the states of the controlled point regarding to the desired trajectories in the second simulation using the Kirchhoff model.

**FIGURE 5:**
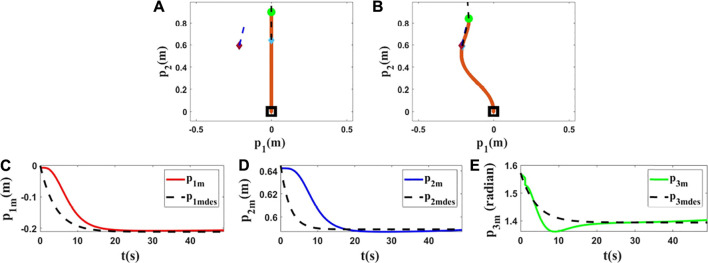
Simulation 3. First row: **(A)** and **(B)** are the initial and final shape of the object, respectively. The desired state of the point *m* is shown as a purple diamond (position) and a dashed line segment (orientation). *L* is 0.90(*m*), *M*_0_ = [0.0000, 0.6430, 1.5708]^*T*^, and *M*_*fdes*_ = [−0.2110, 0.5896, 1.3948]^*T*^. After applying the proposed controller, the final state of *M* is *M*_*f*_ = [−0.2089, 0.5891, 1.4010]^*T*^. Second row: **(C)**, **(D)** and **(E)** are the states of the controlled point regarding to the desired trajectories in the third simulation using the Kirchhoff model.

In the first step of this part, we have chosen the Kirchhoff elastic rod formalism (Bretl and McCarthy, 2014; Shah and Shah, 2016) to use in our tests. Various conditions have been used in each simulation to show the performance of the proposed methodology. *L*, initial position and final position of the rod have been changed in each simulation, and one can find the results in Figures 3–5. *p*_1_ and *p*_2_ are measured in meters and *p*_3_ in radians. In the first simulation, *L* is set to 0.7(*m*), and the initial position and target of the controlled point are set as shown in Figure 3. In the next simulations, we changed *L* to 0.9(*m*) to provide different tests with different conditions, and we applied the proposed method to control the position and orientation of two different controlled points with two different targets. The first row of Figures 3A,B–5A,B displays how the object changed from the initial shape to the final shape in these simulations. The second row of Figures 3C–E–5C–E shows that the proposed strategy can control the manipulated object according to the desired trajectories. Some parameters of ${\hat{C}}_{i}$ for the first simulation are plotted in the third row of Figure 3F,G,H to show how they change during the deformation process.

### 4.2 Simulations Using ARAP Model

**FIGURE 6:**
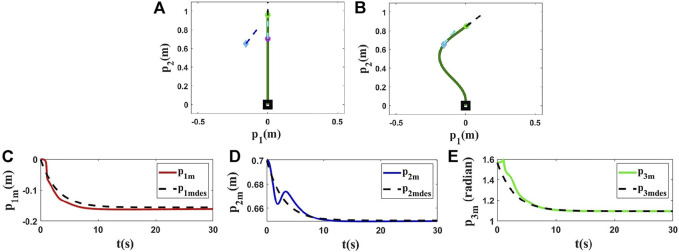
Simulation 4. First row: **(A)** and **(B)** are the initial and final shape of the object, respectively. The desired state of the point *m* is shown as a blue diamond (position) and a dashed line segment (orientation). *L* = 1.00(*m*), *M*_0_ = [0.0000, 0.7000, 1.5708]^*T*^, and *M*_*fdes*_ = [−0.1564, 0.6495, 1.0952]^*T*^. After applying the proposed controller to this model, the final state of the controlled point is [−0.1563, 0.6536, 1.0952]^*T*^. Second row: **(C)**, **(D)** and **(E)** are the states of the controlled point based on the desired trajectories in the fourth simulation using the ARAP model.

**FIGURE 7:**
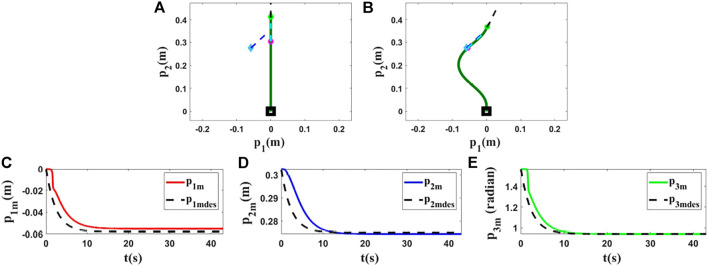
Simulation 5. First row: **(A)** and **(B)** are the initial and final shape of the object, respectively. The desired state of the point *m* is shown as a blue diamond (position) and a dashed line segment (orientation). *L* = 0.43(*m*), *M*_0_ = [0.0000, 0.3000, 1.5708]^*T*^, and *M*_*fdes*_ = [−0.0579, 0.2750, 0.9406]^*T*^. After applying the proposed controller to this model, the final state of the controlled point is [−0.0550, 0.2744, 0.9452]^*T*^. Second row: **(C)**, **(D)** and **(E)** are the states of the controlled point based on the desired trajectories in the fifth simulation using the ARAP model.

**FIGURE 8:**
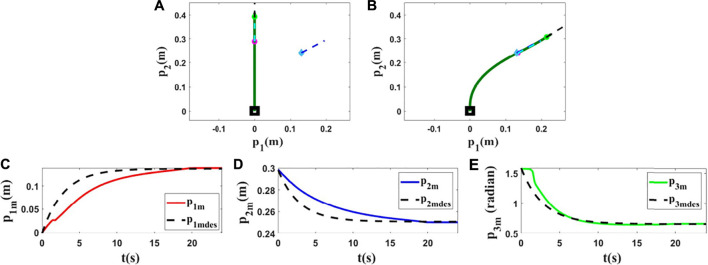
Simulation 6. First row: **(A)** and **(B)** are the initial and final shape of the object, respectively. The desired state of the point *m* is shown as a blue diamond (position) and a dashed line segment (orientation). *L* = 0.43(*m*), *M*_0_ = [0.0000, 0.3000, 1.5708]^*T*^, and *M*_*fdes*_ = [0.1400, 0.2500, 0.6650]^*T*^. After applying the proposed controller to this model, the final state of the controlled point is [0.1415, 0.2502, 0.6673]^*T*^. Second row: **(C)**, **(D)** and **(E)** are the states of the controlled point based on the desired trajectories in the sixth simulation using the ARAP model.

In this part, we use another method to model the studied deformable object. These simulations aim to show that the proposed controller can be used with different models with different dynamic parameters. To do that, we use As-Rigid-As-Possible (ARAP) surface modeling (Sorkine and Alexa, 2007) to model the object. To validate the performance of the proposed controller, three different simulations are conducted, and in each of them, various conditions are applied to the object which means *L*, *M*_0_, and *M*_*fdes*_ have been changed. One can find the results in Figures 6–8. The first row of Figures 6A,B–8A,B displays the initial and final shape of the object in these simulations. The second row of Figures 6C–E–8C–E demonstrates the deformation of the states based on the desired trajectories. It shows that the proposed method can control the object following desired trajectories.

As shown by the various simulations under different conditions (various models, lengths, initial positions, and targets), it is clear that with the proposed method we can move the point to the chosen goal with an acceptable error.

## 5 Robot Experiments

**FIGURE 9:**
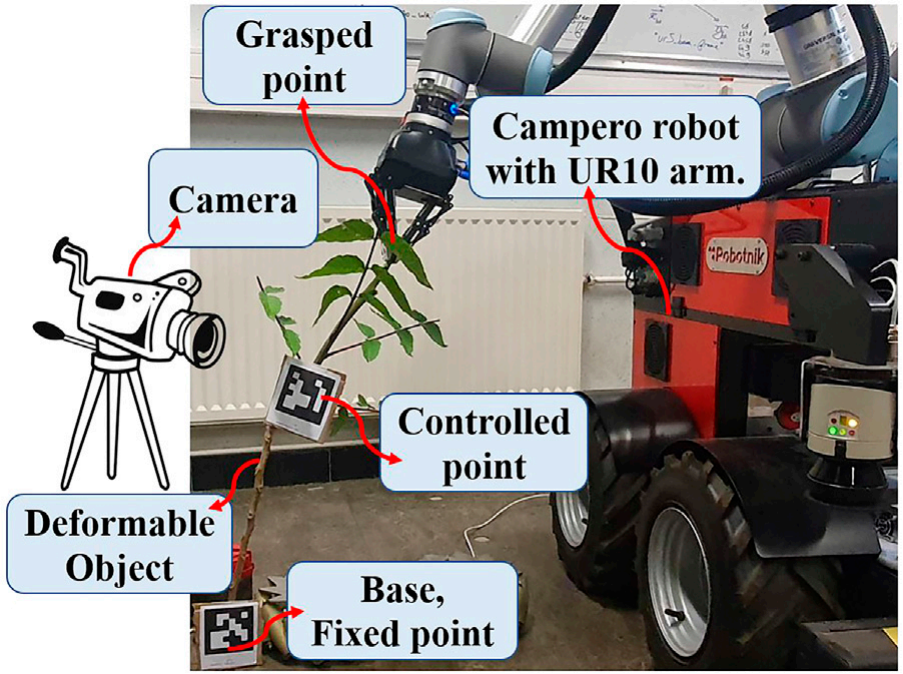
The experimental setup used in this work.

In this section, we conduct different experiments to show the performance of the proposed adaptive control strategy on real objects. The experiments are done with a robot named Campero, consisting of a mobile base and a UR10 arm. The object is rigidly grasped by the robotic gripper, and it is tracked with a fixed camera using Aruco markers. The object’s shape is captured by a Logitech C270 camera using OpenCV. The whole experimental setup is found in Figure 9.

### 5.1 Experiments With a Sponge Rod

**FIGURE 10:**
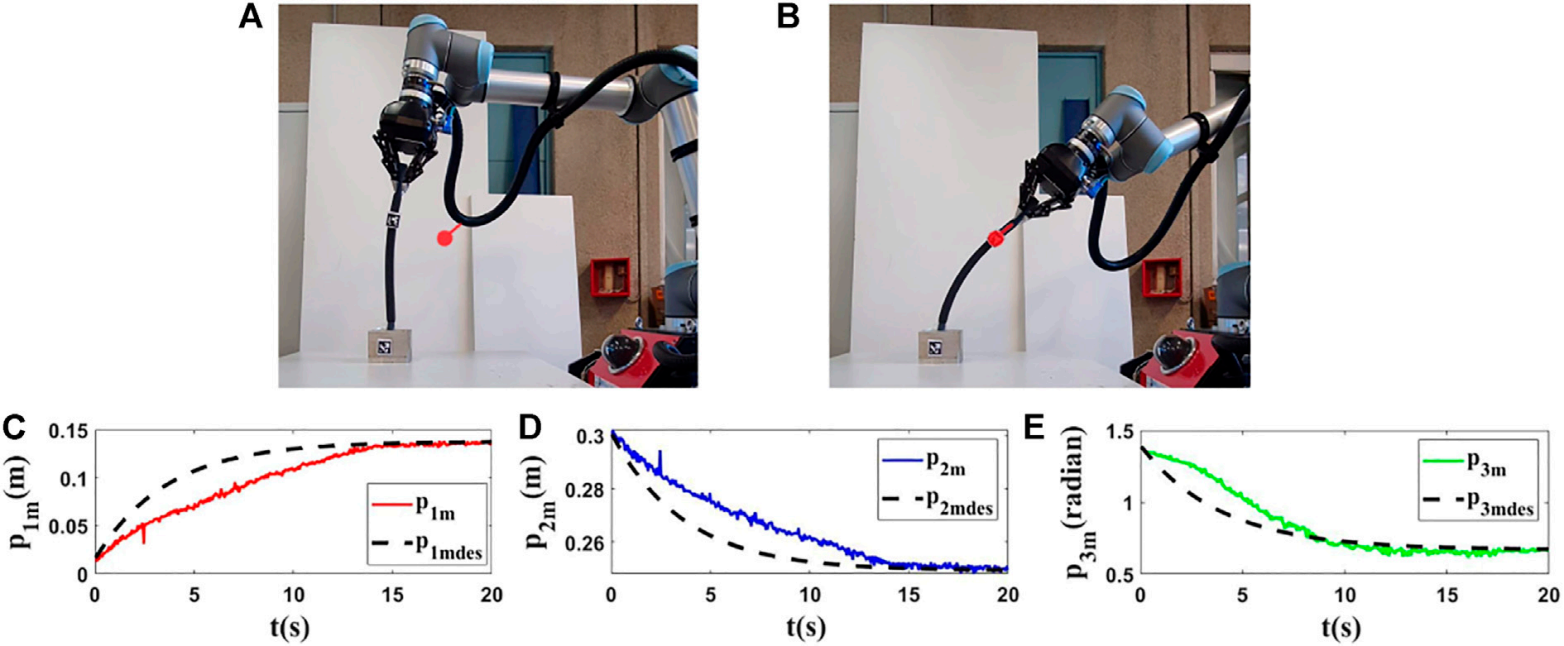
Experiment 1. First row: **(A)** and **(B)** are the initial and final shape of the object, respectively. The desired state of *m* is shown as a red circle with a small line segment. In this test, we assume that *M*_0_ is [0.016,0.300,1.428]^*T*^ and we want to move it to *M*_*fdes*_ = [0.140,0.250,0.665]^*T*^. The final state of the controlled point is *M*_*f*_ = [0.137,0.247,0.661]^*T*^. Second row: **(C)**, **(D)** and **(E)** are the evolution of the states in the first experiment with respect to their desired trajectories.

**FIGURE 11:**
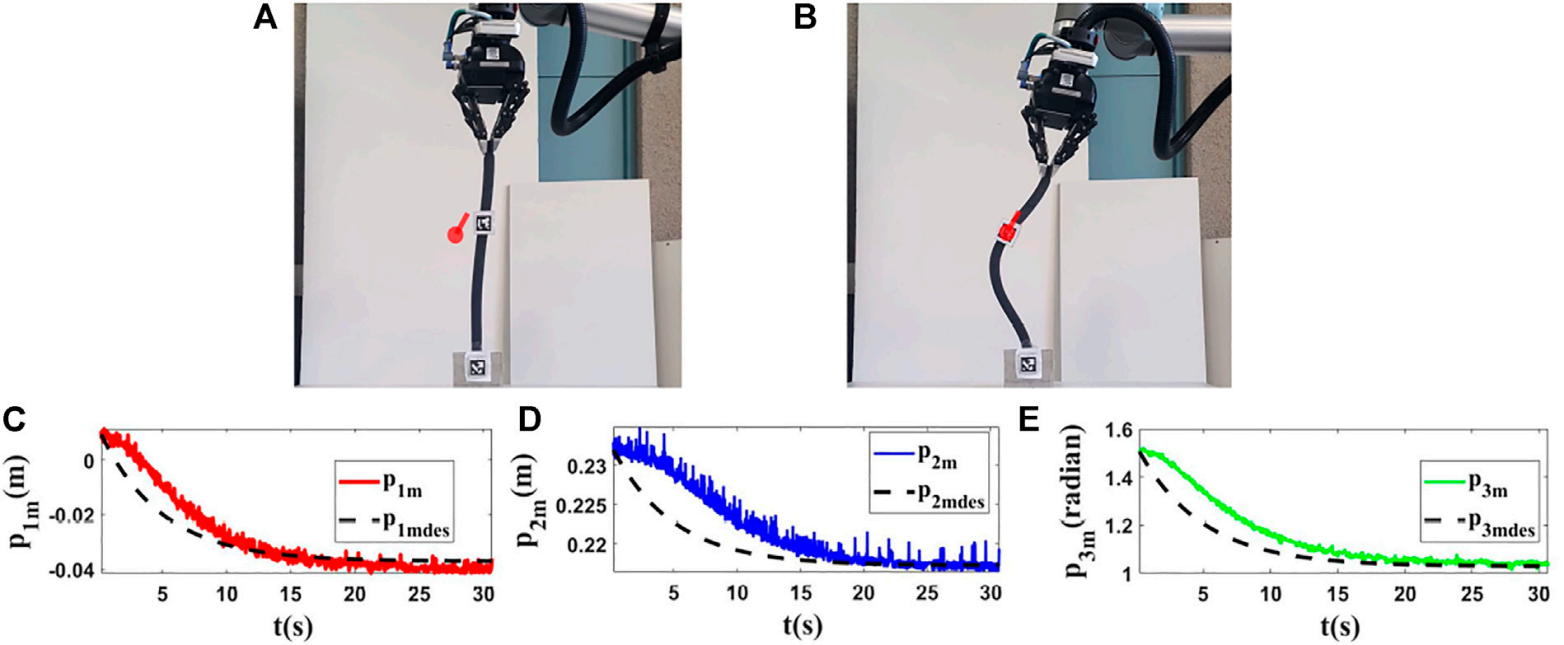
Experiment 2. First row: **(A)** and **(B)** are the initial and final shape of the object, respectively. The desired state of *m* is shown as a red circle with a small line segment. For this test, we assume that *M*_0_ is [0.010,0.232,1.507]^*T*^ and we want to move it to *M*_*fdes*_ = [−0.036,0.217,1.030]^*T*^. The final state of the controlled point is *M*_*f*_ = [−0.039,0.216,1.043]^*T*^. Second row: **(C–E)** are the evolution of the states in the second experiment with respect to their desired trajectories.

For the first and second experiments, we use a sponge rod (*L* = 0.43(*m*)) which has high flexibility. In this part, two different controlled points and two different targets are set to validate the proposed methodology. We use the same target in the first experiment as in the last simulation since the object’s length is the same. With this test, we aim to show that we can expect almost the same results in simulations and experiments for similar conditions. The obtained results (Figures 8, 10) confirm that we obtain almost the same behavior in experiment and simulation. In the second experiment, we set another target and initial position for the controlled point to show the performance of the used method in different conditions. In the first row of Figures 10A,B, 11A,B, the initial and final configuration of the object can be seen. The second row of Figures 10C–E, 11C–E, shows the evolution of the states in these experiments.

### 5.2 Experiments With a Plexiglass Rod

**FIGURE 12:**
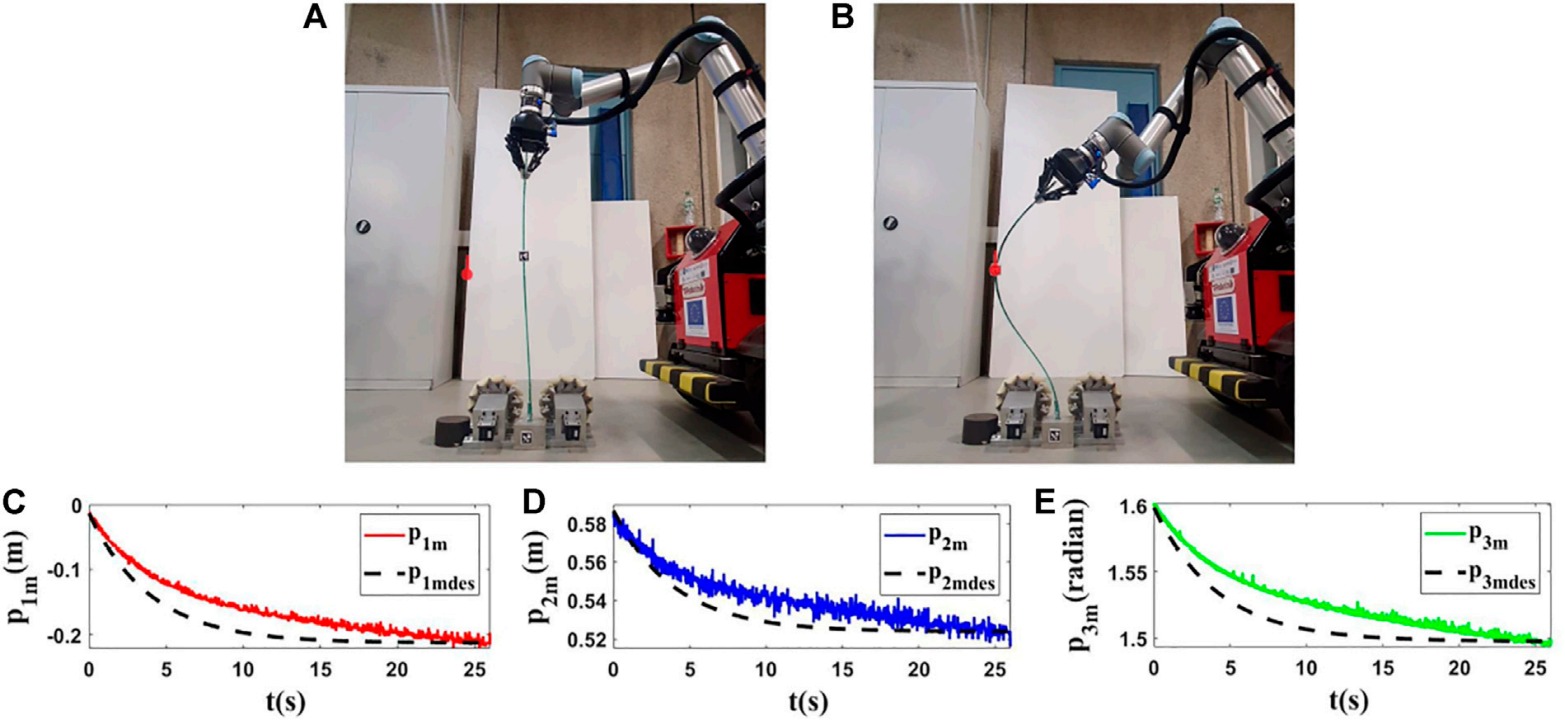
Experiment 3. First row: **(A)** and **(B)** are the initial and final shape of the object, respectively. The desired state of *m* is shown as a red circle with a small line segment. We assume that *M*_0_ is [−0.016,0.585,1.608]^*T*^ and we want to move it to *M*_*fdes*_ = [−0.211,0.523,1.498]^*T*^. The final state of the controlled point is *M*_*f*_ = [−0.209,0.519,1.501]^*T*^. Second row: **(C–E)** are the states of the controlled point in the third experiment with respect to their desired trajectories.

**FIGURE 13:**
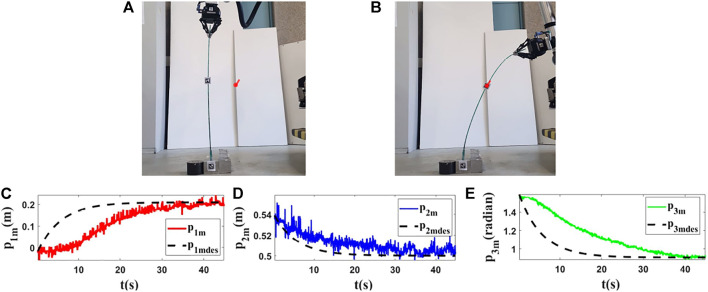
Experiment 4. First row: **(A)** and **(B)** are the initial and final shape of the object, respectively. The desired state of *m* is shown as a red circle with a small line segment. We assume that *M*_0_ is [−0.019,0.539,1.578]^*T*^ and we want to move it to *M*_*fdes*_ = [0.209,0.500,0.908]^*T*^. The final state of the controlled point is *M*_*f*_ = [0.202,0.503,0.908]^*T*^. Second row: **(C–E)** are the states of the controlled point in the fourth experiment with respect to their desired trajectories.

For the third and fourth experiments, we use a deformable linear object made of plexiglass (*L* = 0.95(*m*)). In this section, our objective is to show that the proposed method can work with different objects having different characteristics. The initial and final shapes of the object in these experiments are found in the first row of Figures 12A,B, 13A,B. The second row of Figures 12C,D,E, 13C,D,E displays how the states are changed during these tests.

### 5.3 Experiments With Real Vegetables

For the last experiments, we use two different real vegetables and we do the tests with them. Our goal is to show that the proposed method can be used with natural agricultural rods (vegetable branches) without knowing their properties.

**FIGURE 14:**
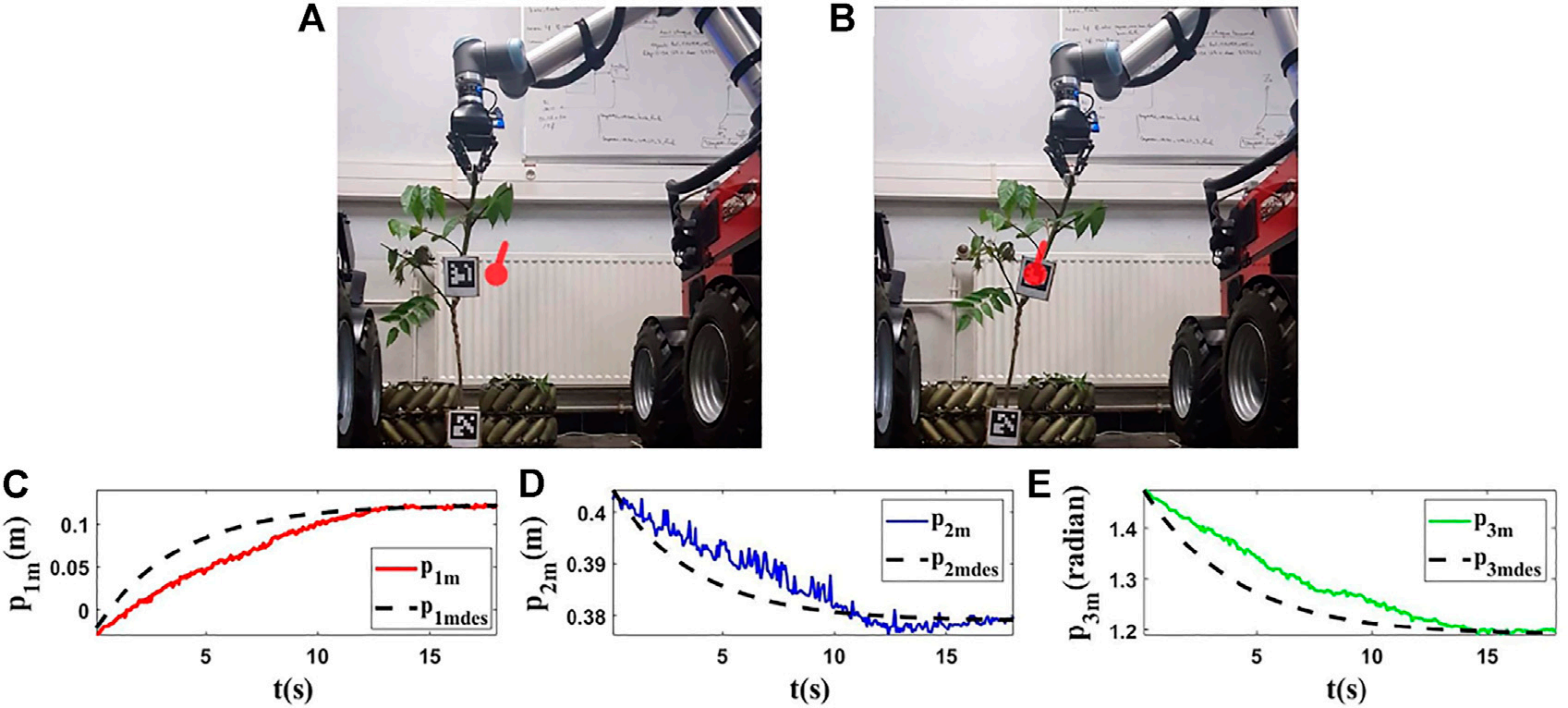
Experiment 5. First row: **(A)** and **(B)** are the initial and final shape of the object, respectively. The desired state of *m* is shown as a red circle with a small line segment. Object’s length (*L*) is 0.72(*m*). We assume that *M*_0_ is [−0.025,0.405,1.480]^*T*^ and we want to move it to *M*_*fdes*_ = [0.123,0.380,1.200]^*T*^. The final state of the controlled point is *M*_*f*_ = [0.122,0.377,1.197]^*T*^. Second row: **(C–E)** are the evolution of the states in the fifth experiment with respect to their desired trajectories.

**FIGURE 15:**
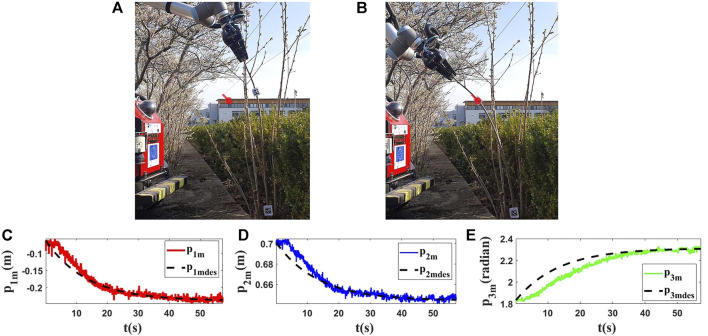
Experiment 6. First row: **(A)** and **(B)** are the initial and final shape of the object, respectively. The desired state of *m* is shown as a red circle with a small line segment. Object’s length (*L*) is 0.99(*m*). We assume that *M*_0_ is [−0.062,0.702,1.833]^*T*^ and we want to move it to *M*_*fdes*_ = [−0.237,0.645,2.306]^*T*^. The final state of the controlled point is *M*_*f*_ = [−0.241,0.646,2.309]^*T*^. Second row: **(C)**, **(D)** and **(E)** are the evolution of the states in the last test with respect to their desired trajectories.

The initial and final shapes of the objects are presented in the first row of Figures 14A,B, 15A,B. Second row of Figures 14C,D,E, 15C,D,E demonstrates the evolution of the states of the controlled points based on the desired trajectories in these experiments. As shown by the results (Figures 14, 15), with the proposed method, the controlled point on the object can reach its target with a tolerable error.

The aforementioned results (Figures 10–15) show that the proposed strategy can control the object in a precise way, and with this method, one can manipulate the taken object desirably following the desired trajectories.

### 5.4 Mean Squared Error

In this section, we will present the mean squared error (MSE) of all experiments to show the performance of the method. MSE measures the average of the squares of the errors between the desired values and the final values. Note that the errors of all experiments are in the same range. Accordingly, we introduce MSE as:

$$e_{v}=\sqrt{\frac{1}{n}\overset{n}{\underset{r=1}{\sum }}{\left(v_{rmf}-v_{rm-fdes}\right)}^{2}}$$ (28)

*n* is the number of experiments (*n* = 6), *v*_*rmf*_ is the final value of each state (*p*_*im*_) of the controlled point and *v*_*rm*−*fdes*_ is the desired final values of them in experiment *r*. Therefore based on the presented result in the previous section, we have:

$$e_{p_{1}}=0.004\left(m\right),\;e_{p_{2}}=0.003\left(m\right),\;e_{p_{3}}=0.007\left(rad\right)$$ (29)

As can be seen, the proposed approach succeeds in controlling very accurately the position and orientation of a point on a deformable rod, despite the different properties of the object.

## Conclusion

In this paper, we have proposed a novel approach to address the problem of controlling a deformable object. An adaptive control method was developed to move an arbitrary point to a chosen objective without knowing prior model parameters. We analyzed the stability of the controlled system via the Lyapunov theory and presented the adaptation law to update the estimations of the system parameters and the states of the controlled point during the control process. The controller was evaluated using Kirchhoff’s elastic rod and ARAP models in simulation. It was shown that the proposed method is not attached to a specific model with predefined parameters and structures. It was also successfully validated using various objects with different characteristics and flexibility in real experiments. For future steps, we will attempt to extend the methodology to control the whole shape of the object and extend the approach in three dimensions.

## Data Availability

The raw data supporting the conclusion of this article will be made available by the authors, without undue reservation.

## Footnotes

1 The video of the simulations and experimental results can be found at: https://www.youtube.com/watch?v=mMsRmehdcXo
